# The Prevalence and Development of Neck and Lower Back Pain and Associated Factors in Dentistry Students—A Long-Term Prospective Study

**DOI:** 10.3390/ijerph19148539

**Published:** 2022-07-13

**Authors:** Lenka Hodačová, Nela Pilbauerová, Eva Čermáková, Stanislav Machač, Jan Schmidt, Jan Hodač, Martin Kapitán

**Affiliations:** 1 Department of Preventive Medicine, Charles University, Faculty of Medicine in Hradec Králové, 500 03 Hradec Králové, Czech Republic; hodacja@lfhk.cuni.cz; 2 Department of Dentistry, Charles University, Faculty of Medicine in Hradec Králové, University Hospital Hradec Králové, 500 05 Hradec Králové, Czech Republic; nela.pilbauerova@lfhk.cuni.cz (N.P.); jan.schmidt@lfhk.cuni.cz (J.S.); kapitanm@lfhk.cuni.cz (M.K.); 3 Department of Medical Biophysics, Charles University, Faculty of Medicine in Hradec Králové, 500 03 Hradec Králové, Czech Republic; cermakovae@lfhk.cuni.cz; 4 Department of Rehabilitation and Sports Medicine, Charles University, Second Faculty of Medicine, University Hospital Motol, 150 06 Prague, Czech Republic; machac.s@seznam.cz; 5 Institute of Sports Medicine, 150 00 Prague, Czech Republic

**Keywords:** musculoskeletal disorders, dentistry students, multivariate analysis, neck pain, lower back pain, risk factors

## Abstract

Musculoskeletal disorders (MSDs) represent a significant occupational burden for dentists and dentistry students. This study aimed to assess the five-year development of most common musculoskeletal complaints among dentistry students during the whole length of their studies and to analyze the impact of some potentially associated risk factors. A longitudinal questionnaire survey regarding the occurrence of MSDs was conducted among a total of 73 dentistry students between 2014 and 2021. The same students enrolled in three consecutive years were monitored throughout their entire studies. Statistical analysis was performed using methods of descriptive statistics, Chi-square test, Fisher’s exact test, McNemar test, and univariate and multivariate logistic regression analyses. The most frequent areas of complaints were neck (61.6%) and lower back (49.3%). The increase in self-reported complaints between the first and the fifth year was statistically significant for neck pain (increase by 15%, *p* = 0.043) but not for lower back pain (by 6.8%, *p* = 0.44). According to our data, age, performing top-level sport, and participating in regular sporting activities had a statistically significant influence on neck and/or back pain. The results of this longitudinal prospective study showed that neck, as well as lower back pain, are significant problems for dentistry students.

## 1. Introduction

Dentistry is a demanding profession, both physically and mentally [1]. Despite considerable progress and numerous technical improvements in recent years, many work-related health issues still exist in modern dentistry [2,3,4]. Musculoskeletal disorders (MSDs) are among the common problems associated with the dental profession, as confirmed by the results of numerous studies [5,6,7]. MSDs frequently occur among the present adult population and have a considerable and growing impact worldwide. In the Czech Republic, MSDs have long been one of the most common causes of sick leave as well as disability [8,9].

In dentistry, among the factors that lead to increasing risks of MSDs are repetitive, poor, and long-term work habits, such as awkward static neck and back positions, repeated muscle movements of the upper limb and wrists, high risk of muscle strains, isometric muscle contractions, small working field, working with vibrating devices as well as psychological demands and stress [10,11,12,13,14,15,16,17,18]. With the onset of the COVID-19 pandemic, the use of other protective tools has become necessary. This led to increased risks of imbalanced postures and reduced freedom of movements. Such worsened work conditions, in combination with the awareness of high risk of infection transmission through an extensive production of aerosol, increased mental stress in dentists [19]. Lack of physical activity [20,21], age, genetic predispositions [22,23], female gender [21,24,25,26,27,28], obesity, and smoking [29] are some of the other risk factors associated with increased risk of MSDs.

The literature reports that the most commonly affected body areas in dentists are lumbar region, neck, shoulders, hands and wrists [5,7,20,30,31,32,33]. However, some studies confirmed that MSDs have an early onset and the problems frequently start during undergraduate dentistry studies [14,15,33]. Several papers showed that the occurrence of MSDs is higher in the latter phases of dentistry studies. These studies compared actual students in different years, i.e., groups of different persons. Rising et al. found higher frequency and daily duration of pain in the third-year students compared with the first-year students [22]. Khan and Chew [27] reported more MSDs among students in the clinical phase of their studies. To the authors’ knowledge, there are no studies in the available literature that would follow one particular cohort of undergraduate dentistry students throughout the complete running of their studies and assess changes, in particular most common self-reported MSD complaints and the risk factors that could influence them.

The aim of this study was to assess the five-year development, prevalence, and intensity of the most common musculoskeletal complaints among dentistry students during the full length of their studies, as well as to analyze the impact of some potentially associated risk factors. We build on our previous article, which dealt with the analyses of self-reported overall MSDs in the same sample [34]. This paper focuses on MSDs in the individual body regions.

## 2. Materials and Methods

A longitudinal prospective study was conducted through a self-administered, structured questionnaire. Participation was voluntary, and all the students willing to participate signed informed consent. The methods of this research were approved by the Dean of the Charles University, Faculty of Medicine in Hradec Králové and the Ethics Committee of the University Hospital Hradec Králové (Ref. no. 201410 S04P).

All the dentistry students newly enrolled at the Charles University, Faculty of Medicine in Hradec Králové, the Czech Republic were contacted and asked to participate in the study. Enrollment in one of the included academic years (2014/15, 2015/16, and 2016/17) was the only inclusion criterion. No exclusion criteria were applied at the entry phase of the study. During the follow-up, we excluded students with an irregular running of their studies (repeated year, individual study plan, an internship abroad, etc.).

A control group consisted of general medicine students; the same inclusion (general medicine study program enrollment) and exclusion criteria were applied. Unfortunately, a significant number of general medical students did not complete the five-year follow-up. Therefore, for the present analysis focusing on the development of the most common musculoskeletal complaints during dentistry studies, only a sample of the dentistry students was used.

The participants filled the same questionnaire at the beginning (first year), in the middle (third year) and at the end (fifth year) of the dentistry/general medicine course. The questionnaire included three sections of a total of 25 closed-ended questions regarding (i) their personal data; (ii) self-reported overall MSDs, possibly associated factors; and (iii) the intensity of pain in 10 individual body areas. A scale of no–mild–moderate–severe pain was used. The questionnaire was based on our previous studies among dentists [6,15], modified for the students and pre-piloted. Detailed analyses of the data from the first two parts of the questionnaire concerning descriptive data and the statistically significant correlation between the occurrence and development of overall MSDs and the risk factors followed are described in our previous paper [34].

The development of self-reported pain and its intensity in the different body regions among the dentistry students was monitored for five years, i.e., between the first and the fifth year. The six most common regions of pain (neck, lower back, head, upper back, shoulders, and wrists/hands) were analyzed using the univariate analyses. The intensity of pain was dichotomized and analyzed in two categories—no pain and mild/moderate/severe pain. The results in four body areas (head, upper back, shoulders, and wrists/hands) were either without statistical significance or were inconsistent. As such, they would not allow relevant conclusions to be drawn. Therefore, only the two most common regions of problems, i.e., neck and lower back pain, were selected for further multivariate analysis. The results are presented in this paper.

Statistical analysis was performed using NCSS 2019 Statistical Software (NCSS, LLC, Kaysville, UT, USA, ncss.com/software/ncss; accessed on 1 June 2021) through methods of descriptive statistics, Chi-square test, Fisher’s exact test, and McNemar test. Univariate and multivariate logistic regression analysis was performed to evaluate the association between the followed factors and the most frequently common areas of musculoskeletal pain. The level of statistical significance was set to α = 0.05.

## 3. Results

**Figure 1:**
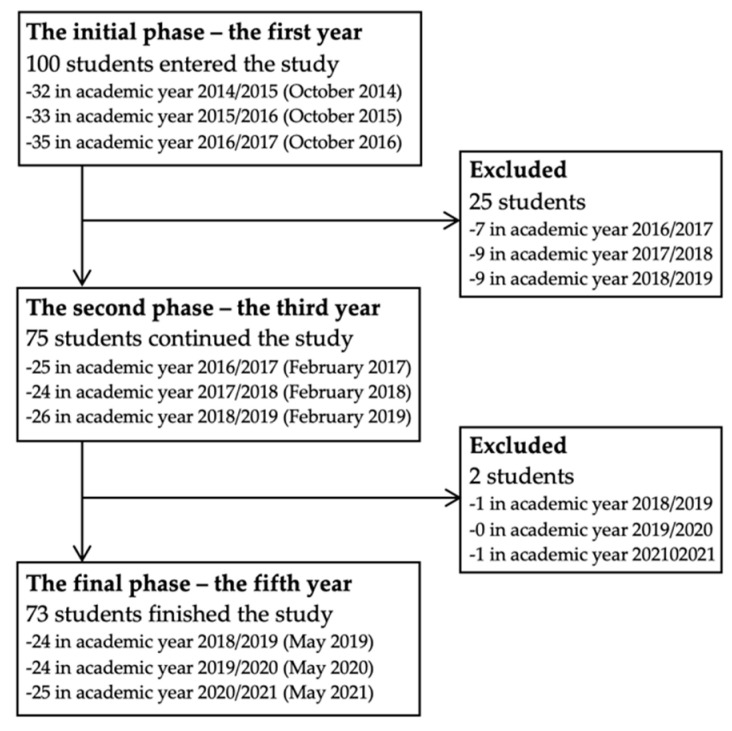
The flow diagram of the number of respondents participating and excluded during the running of the study.

A total of 100 dentistry students were addressed and included in the first phase of the study. Unfortunately, some of them were excluded because they met the exclusion criteria or refused to continue in the study. Finally, 73 students (73%) remained and were monitored throughout the whole period of their studies, i.e., for five years. Changes in the number of participants during the running of the study are shown in Figure 1.

In our sample, 24.7% of the respondents were men (*n* = 18) and 75.3% were women (*n* = 55). The mean age of respondents in the initial phase of the study was 19.6 (±1.1). Height was 181.2 cm (±8.4) in men and 167.9 cm (±5.8) in women. Weight was 75.9 kg (±13.5) in men and 59.1 kg (±6.4) in women in the first year, and it significantly increased between the first and the fifth year both in men (by 3.0 kg; *p* = 0.014) and in women (by 1.7 kg; *p* = 0.00046).

The occurrence and intensity of recent pain in the ten monitored body regions are shown in Table 1. The most frequently reported complaints were neck pain and lower back pain, reported by 61.6% and 49.3% of the fifth-year students, respectively.

**Figure 2:**
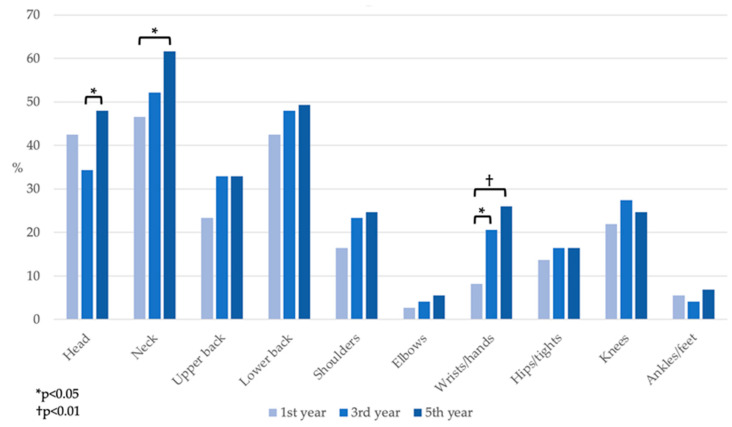
The development of recent pain in different body regions regardless of its intensity.

The summarized data (mild + moderate + severe pain) of the occurrence and development of recent pain in the monitored body regions are demonstrated in Figure 2. In all monitored body parts, an increase in problems can be seen between the first and the fifth year among dentistry students in our sample. Statistically significant differences were found in the occurrence of headache between the third and fifth year (*p* = 0.041), neck pain between the first and the fifth (*p* = 0.043), and wrists/hands pain between first and third (*p* = 0.035) and first and fifth (*p* = 0.0023) year.

The results of univariate analysis for neck and lower back pain are presented in Table 2 and Table 3.

The univariate analysis found a statistically significant influence on neck pain only for the top-level sport participation in the third and fifth year and of the regular sporting activity in the fifth year. The students’ age and the women’s weight were statistically significantly associated with lower back pain in the fifth year. Top-level sport and weight of men statistically significantly influenced the risk of back pain in the first year. The association between the diseases of the musculoskeletal system in blood relatives and the occurrence of back pain was statistically significant among third-year students.

The multivariate analysis confirmed the statistically significant influence of top-level sport and regular sporting activity on the occurrence of neck pain. For neck pain, the whole model was statistically significant in the fifth year. For lower back pain, a significant association was found for height, actual weight, and top-level sport activity in the first year; diseases of the musculoskeletal system in blood relatives in the third year; age, and awareness of MSDs among dentists in the fifth year. The detailed results of the multivariate analysis on risk factors for the occurrence of neck and lower back pain are shown in Table 4 and Table 5.

## 4. Discussion

Economically active people, in general, spend approximately one-third of their life at the workplace, and almost one-third of workers are exposed to significant occupational risks. Based on the World Health Organization data, work-related health problems lead to significant economic losses of around 4–6% of GDP for most countries [35,36]. Work-related MSDs are also responsible for the disability and early retirement of dentists [17,18]. As MSDs begin early even in undergraduate dentistry students, it is therefore important to address this issue and try to reveal which areas of the human body are the most problematic, which factors lead to worsening pain, and thus how to prevent them. This may contribute to the endeavor to find effective preventive measures.

Our study aimed to monitor the development of the most frequent musculoskeletal complaints in various human body areas among dentistry students throughout their entire studies. Contrary to our previously published paper [34], which presented the results of the analysis of subjectively declared overall musculoskeletal disorders and related factors, this article focuses on musculoskeletal pain in ten followed body regions. The main group of participants, i.e., dentistry students, was the same. The control group, i.e., general medicine students, was omitted because its size significantly decreased during the running of the study.

Consistently with other studies among dentists and dentistry students, the most frequent painful body parts were neck and lower back [3,5,28,30,31,32]. In the fifth year, almost two-thirds (62%) of our students reported neck pain, and half of them (49%) reported lower back pain. The increase in self-reported complaints between the first and the fifth year was statistically significant for neck pain (increase by 15%) but not for lower back pain (by 6.8%). Pain intensity was mostly reported as mild or moderate in both monitored body parts. Only one student reported severe lower back pain.

Thornton et al. [23] reported an occurrence of pain among second-, third-, and fourth-year dentistry students. Pain of the neck, back, shoulders, and hands was declared by 48%, 44%, 31%, and 20% of them, respectively. The occurrence of pain was a bit lower in the neck and back, similar in the hands, and greater in the shoulders, compared to our third-year students.

In the study of Rising et al. [22], 29.41% of male first-year students and 50% of female suffered from neck/shoulder pain; in the third year, it was 58.06% and 47.83% of male and female dentistry students, respectively. Lower back pain was reported by 17.65% and 25.0% of male and female students in the first year, respectively, and by 32.26% and 43.48% in the third year. Our results showed higher occurrence of pain in both these body regions in both first- and third-year students.

Diaz-Caballero et al. [25] demonstrated an 80% occurrence of musculoskeletal pain among dentistry students from VIII to X semesters. The most frequent areas of pain were neck, lower back (15% both), upper back, mid back, wrists (13% all), forearm, and arm (6% both). Compared to our fifth-year students, the overall occurrence of pain was higher, whereas the frequency of pain in the individual body regions was smaller.

Khan and Chew [27] found that students in the clinical years suffered from pain more than students in non/clinical years. The “neck and upper back” pain occurrence was 41% and 82% in the non-clinical and clinical students, respectively; lower back pain was declared by 28% and 64% of the non-clinical and clinical students, respectively. The clinical students declared more “neck and upper back” and “lower back” pain than our fifth-year students.

The study by Ng. et al. [28] showed a 57.7% and 64% prevalence of neck pain and lower back pain, respectively, among the final year dentistry students, whereas in the first year, it was 33.3% in both these regions. Our first-year students suffered from pain in both these regions more, whereas, in the fifth year, they reported lower back pain less, but more neck pain.

In comparison with the study of Czech dentists [6,15], the level of prevalence and intensity of both neck and lower back pain among students was lower, as expected. A total of 78.1% and 75.0% of dentists suffered from neck pain and lower back pain, respectively, regardless of its intensity. The students have spent only a short time performing dentistry work; thus, they were not exposed for a sufficient time to the risk factors contributing to MSDs in the dentistry profession.

The most exposed areas in terms of the development of MSDs are, in general, the cervical and lumbar spine. Bearing in mind that the work of a dentist often requires a head tilt forward of more than 15–20°, it is apparent that this results in overloading the neck muscles and joints of the cervical spine. This position causes the tension of neck extensor muscles as they must hold the head against gravity. Another risk factor is prolonged work with raised arms in static isometric/eccentric contraction. The head tilt, as well as the prolonged work with raised arms, causes muscle tension, leads to neck pain, and can also affect the cervical lordosis curve. Loss of cervical lordosis can cause functional disabilities, pain, or more severe disc protrusion [15,16,20,24,27].

The main reasons leading to work-related musculoskeletal disorders in the lumbar region include loss of lumbar lordosis, mainly due to incorrect sitting position, forward bending at work, and lack of hip tilting in the sitting position. The relative weakness of the lumbar spine stabilizer muscles due to long and incorrect sitting posture also plays a role [2].

In our work, we further monitored and analyzed the impact of some potential risk factors on the development of neck and lower back pain. The reason for the dichotomization of the pain intensity was that only very few students reported moderate or severe intensity of pain, regardless of the followed body region. The literature indicates that musculoskeletal problems in general [7,22,25], as well as neck and back pain, are more frequent in women than in men [14,18,21,33]. The effect of gender on neck pain and lower back pain has not been demonstrated in our sample. Our results are in line with some other studies where the prevalence of back pain was similar for both genders [2,37].

Age influenced the occurrence of back pain in the fifth year only. With the increasing age of students, each additional year of age seems to have a greater impact on the occurrence of back pain.

Top-level sport was explained in our previous article as “a sporting activity that is organized in sport clubs, includes regular workouts, practice, matches, and competitions, is performed on a long-term basis, and the athletes are registered by the official, state-controlled body” [34]. Based on the multivariate analysis, performing top-level sport increased the occurrence of neck pain in the third year more than 11 times and in the fifth year more than 21 times, and the occurrence of lower back pain in the first year more than 9 times. This finding supports the results of our previous analysis, which showed an association between top-level sport and both the occurrence and the development of self-reported overall MSDs [34]. Thus, students who consider studying dentistry and are or were engaged in top-level sport activities should be informed of this burden in advance.

Regular sporting activity was found to be a significant protective factor in the occurrence of neck pain in the fifth year. The influence of regular sporting activity on back pain (in all years) and on neck pain (in the first and the third year) was varying and not statistically significant. Inconsistent answers from the students, unequal distribution of sports activities during the year, and the variable volume of study activities in different parts of the academic year may explain the conflicting results found. Our study was carried out in three phases: the first year in October, the third year in February, and the fifth year in May. There is no general agreement in the scientific community regarding the beneficial influence of sporting activities in connection to MSDs in dentists and dentistry students [2,6,15,24,32].

The important strength of our study is its longitudinal character. The prospective follow-up of the same persons is more reliable than cross-sectional studies comparing different study groups, where the results may be affected by interindividual variability. The authors followed the same group of students for five years, i.e., during their entire studies of dentistry. To the authors’ knowledge, there are no studies in the available literature that would follow the development and changes of the most common self-reported MSDs and risk factors influencing them among one cohort of undergraduate dentistry students throughout the whole length of their studies. Other recent studies published in the literature on a similar topic analyzed only the variances between different groups of students.

Some limitations of the study have also been identified. A certain limit may be a relatively small number of respondents. This could be responsible for some of the inconsistent results in the analysis of the impact of risk factors on the occurrence of MSDs in different body parts. The limited number of respondents may decrease the strength of the statistical analysis. It is important to state that our faculty is relatively small. Only around 35 students are regularly admitted to the study program “Dentistry” each year. Therefore, the authors increased the sample size by observing students from three consecutive study years. Almost all the students entered the study, but unfortunately, some of them were excluded due to meeting the exclusion criteria.

Another limitation is that all the analyses were based on a self-reported questionnaire. This may carry the possibility of various biases. However, questionnaire inquiries are part of the most common methods in general. Questionnaires are used in similar studies of dentists and dentistry students due to being a cheap, fast, and relatively reliable method. The questionnaire was created by a modification of a questionnaire used in previous studies and pre-piloted. The answers can be considered consistent as there were the same respondents and the same questionnaire in all three phases of the study.

Further research is necessary to find more information about the early development of MSDs in different body parts during the very beginning of the dentistry career. Nevertheless, the results of our study can illustrate general trends in the occurrence and development of MSDs in different body parts during dentistry studies. The authors plan to continue to follow the same sample of respondents in the future.

Nowadays, people are very susceptible to MSDs. New technologies and lack of mobility are critical factors for the increasing occurrence of musculoskeletal disorders among young people in general [2,38]. To minimize MSDs among dentistry students and dentists, the identification of controllable variables, early preventive measures, and strategies are recommended at the beginning of their career. As MSDs in dentistry students seem to be a significant problem, universities should focus more on teaching ergonomics guidelines, the correct posture, preventive exercises, and formation and training on body mechanics to develop healthy working habits. Education in and the application of ergonomics should begin early during dentistry studies and continue through the life-long postgraduate education of dentists.

## 5. Conclusions

The occurrence of pain in all body regions increased during the dentistry studies; neck and lower back were the most frequent areas of pain.

Age and performing top-level sport had a negative influence on pain, whereas the effect of regular sporting activity was rather protective.

These results extend our knowledge of MSDs in the early stages of a dentistry career, which can be helpful for potential dentistry students during decision making about their future profession, the motivation of current dentistry students to adopt the ergonomic preventive measures, and the universities when dental ergonomics is educated.

## Figures and Tables

**Table 1:** The occurrence and intensity of recent pain in different body regions.

	1st Year				3rd Year				5th Year			
	No	Mild	Moderate	Severe	No	Mild	Moderate	Severe	No	Mild	Moderate	Severe
	% (*n*)	% (*n*)	% (*n*)	% (*n*)	% (*n*)	% (*n*)	% (*n*)	% (*n*)	% (*n*)	% (*n*)	% (*n*)	% (*n*)
Head	57.5 (42)	28.8 (21)	13.7 (10)	0 (0)	65.8 (48)	26.0 (19)	8.2 (6)	0 (0)	52.1 (38)	34.2 (25)	11.0 (8)	2.7 (2)
Neck	53.4 (39)	32.9 (24)	13.7 (10)	0 (0)	47.9 (35)	41.1 (30)	11.0 (8)	0 (0)	38.4 (28)	41.1 (30)	20.5 (15)	0 (0)
Upper back	76.7 (56)	17.8 (13)	5.5 (4)	0 (0)	67.1 (49)	24.7 (18)	8.2 (6)	0 (0)	67.1 (49)	24.7 (18)	6.8 (5)	1.4 (1)
Lower back	57.5 (42)	38.4 (28)	4.1 (3)	0 (0)	52.1 (38)	39.7 (29)	8.2 (6)	0 (0)	50.7 (37)	38.4 (28)	9.6 (7)	1.4 (1)
Shoulders	83.6 (61)	12.3 (9)	2.7 (2)	1.4 (1)	76.7 (56)	16.4 (12)	6.8 (5)	0 (0)	75.3 (55)	20.5 (15)	2.7 (2)	1.4 (1)
Elbows	97.3 (71)	2.7 (2)	0 (0)	0 (0)	95.9 (70)	2.7 (2)	1.4 (1)	0 (0)	94.5 (69)	4.1 (3)	1.4 (1)	0 (0)
Wrists/hands	91.8 (67)	5.5 (4)	1.4 (1)	1.4 (1)	79.5 (58)	16.4 (12)	4.1 (3)	0 (0)	74.0 (54)	23.3 (17)	1.4 (1)	1.4 (1)
Hips/tights	86.3 (63)	11.0 (8)	2.7 (2)	0 (0)	83.6 (61)	12.3 (9)	4.1 (3)	0 (0)	83.6 (61)	12.3 (9)	0 (0)	4.1 (3)
Knees	78.1 (57)	15.1 (11)	4.1 (3)	2.7 (2)	72.6 (53)	23.3 (17)	4.1 (3)	0 (0)	75.3 (55)	19.2 (14)	4.1 (3)	1.4 (1)
Ankles/feet	94.5 (69)	4.1 (3)	1.4 (1)	0 (0)	95.9 (70)	2.7 (2)	1.4 (1)	0 (0)	93.2 (68)	5.5 (4)	1.4 (1)	0 (0)

**Table 2:** The univariate analysis of the influence of the followed factors on the occurrence of neck pain.

	1st Year		3rd Year		5th Year	
	OR (95% CI)	*p*	OR (95% CI)	*p*	OR (95% CI)	*p*
Gender (men)	1.12 (0.38, 3.27)	0.83	2.03 (0.68, 6.02)	0.2	2.57 (0.87, 7.62)	0.087
Age	1.18 (0.76, 1.83)	0.45	1.15 (0.77, 1.71)	0.49	1.20 (0.77, 1.87)	0.4
Height	1.00 (0.95, 1.06)	0.98	0.97 (0.93, 1.04)	0.63	0.98 (0.93, 1.04)	0.53
Height—men	1.05 (0.93, 1.19)	0.39	1.04 (0.92, 1.17)	0.54	1.04 (0.93, 1.17)	0.46
Height—women	0.98 (0.90, 1.08)	0.69	1.00 (0.92, 1.10)	0.92	1.01 (0.92, 1.11)	0.86
Actual weight—men	1.04 (0.96, 1.12)	0.35	1.04 (0.96, 1.12)	0.34	1.04 (0.97, 1.13)	0.25
Actual weight—women	1.00 (0.92, 1.09)	0.94	1.02 (0.94, 1.10)	0.61	1.02 (0.94, 1.10)	0.71
Weight increase			0.71 (0.25, 2.04)	0.73	1.14 (0.41, 3.19)	0.94
Weight decrease			1.20 (0.32, 4.44)		0.85 (0.17, 4.33)	
General disease	1.64 (0.44, 6.18)	0.46	0.57 (0.15, 2.15)	0.4	1.85 (0.60, 5.67)	0.28
Chronic medication	1.61 (0.55, 4.71)	0.38	1.05 (0.35, 3.11)	0.93	0.38 (0.11, 1.33)	0.12
Dominant hand (right)	0.63 (0.13, 3.01)	0.56	1.09 (0.21, 5.81)	0.92	1.68 (0.31, 8.97)	0.55
Diseases of musculoskeletal system in blood relatives	0.92 (0.37, 2.32)	0.86	1.12 (0.43, 2.92)	0.82	0.81 (0.31, 2.12)	0.67
Past disease or trauma of musculoskeletal system	1.60 (0.33, 7.72)	0.56	0.90 (0.28, 2.90)	0.86	1.30 (0.35, 4.79)	0.69
Top-level sport	2.29 (0.73, 7.17)	0.15	5.89 (1.19, 29.17)	0.013	9.82 (1.20, 80.37)	0.0059
Regular sporting activities	2.28 (0.70, 7.40)	0.16	1.10 (0.29, 4.18)	0.89	0.19 (0.04, 0.92)	0.018
Awareness of MSDs among dentists	1.17 (0.46, 2.93)	0.74	1.48 (0.59, 3.73)	0.4	0.88 (0.34, 2.25)	0.78
Considering the range of ergonomic education as sufficient			1.11 (0.35, 3.55)	0.86	0.67 (0.21, 2.19)	0.5

OR, odds ratio; CI, confidence interval.

**Table 3:** The univariate analysis of the influence of the followed factors on the occurrence of lower back pain.

	1st Year		3rd Year		5th Year	
	OR (95% CI)	*p*	OR (95% CI)	*p*	OR (95% CI)	*p*
Gender (men)	1.67 (0.55, 5.08)	0.36	1.21 (0.41, 3.51)	0.73	0.96 (0.33, 2.80)	0.95
Age	1.02 (0.67, 1.55)	0.92	1.37 (0.87, 2.16)	0.14	2.29 (1.23, 4.29)	0.0021
Height	0.97 (0.92, 1.02)	0.26	0.96 (0.90, 1.01)	0.13	1.00 (0.95, 1.05)	0.97
Height—men	1.08 (0.94, 1.24)	0.23	0.98 (0.87, 1.09)	0.67	1.09 (0.96, 1.24)	0.16
Height—women	0.92 (0.83, 1.01)	0.074	0.92 (0.83, 1.01)	0.068	0.95 (0.86, 1.06)	0.22
Actual weight—men	1.09 (0.99, 1.20)	0.047	0.99 (0.92, 1.06)	0.8	1.01 (0.95, 1.09)	0.7
Actual weight—women	0.98 (0.90, 1.06)	0.6	0.95 (0.88, 1.03)	0.2	0.92 (0.85, 1.00)	0.045
Weight increase			0.60 (0.21, 1.76)	0.23	0.68 (0.25, 1.84)	0.7
Weight decrease			2.11 (0.54, 8.16)		0.65 (0.13, 3.27)	
General disease	1.35 (0.36, 5.09)	0.65	0.73 (0.20, 2.65)	0.63	0.70 (0.23, 2.14)	0.53
Chronic medication	1.50 (0.51, 4.37)	0.46	0.95 (0.32, 2.83)	0.93	1.54 (0.44, 5.41)	0.49
Dominant hand (right)	0.98 (0.20, 4.74)	0.98	0.43 (0.07, 2.51)	0.33	2.06 (0.35, 12.02)	0.41
Diseases of musculoskeletal system in blood relatives	0.63 (0.25, 1.62)	0.34	0.33 (0.12, 0.91)	0.027	1.86 (0.72, 4.82)	0.2
Past disease or trauma of musculoskeletal system	1.93 (0.40, 9.31)	0.41	1.11 (0.35, 3.55)	0.86	1.54 (0.44, 5.41)	0.49
Top-level sport	4.07 (1.24, 13.36)	0.016	0.48 (0.13, 1.78)	0.26	1.83 (0.54, 6.24)	0.33
Regular sporting activities	1.30 (0.42, 4.07)	0.65	0.91 (0.24, 3.45)	0.89	1.14 (0.37, 3.56)	0.82
Awareness of MSDs among dentists	0.66 (0.26, 1.67)	0.38	0.90 (0.30, 2.73)	0.85	1.33 (0.44, 4.05)	0.61
Considering the range of ergonomic education as sufficient			0.90 (0.28, 2.90)	0.86	0.97 (0.32, 2.93)	0.95

OR, odds ratio; CI, confidence interval.

**Table 4:** The multivariate analysis of the influence of the followed factors on the occurrence of neck pain.

	1st Year		3rd Year		5th Year	
	OR (95% CI)	*p*	OR (95% CI)	*p*	OR (95% CI)	*p*
Gender (men)	1.33 (0.24, 7.27)	0.74	2.74 (0.41, 18.14)	0.28	6.98 (0.65, 74.40)	0.08
Age	1.09 (0.66, 1.78)	0.74	1.06 (0.65, 1.73)	0.81	1.15 (0.68, 1.94)	0.59
Height	0.99 (0.88, 1.10)	0.81	0.92 (0.82, 1.04)	0.18	0.95 (0.85, 1.08)	0.45
Actual weight	1.01 (0.93, 1.10)	0.75	1.08 (0.99, 1.18)	0.073	1.07 (0.96, 1.19)	0.21
Weight increase			0.58 (0.15, 2.18)	0.67	1.71 (0.43, 6.74)	0.73
Weight decrease			1.17 (0.26, 5.29)		0.92 (0.10, 8.81)	
General disease	3.26 (0.59, 18.06)	0.16	0.26 (0.03, 2.08)	0.19	1.23 (0.13, 11.62)	0.86
Chronic medication	2.75 (0.64, 11.91)	0.17	0.38 (0.07. 2.10)	0.26	0.39 (0.03, 4.34)	0.44
Dominant hand (right)	0.53 (0.08, 3.58)	0.51	1.83 (0.20, 16.59)	0.59	4.23 (0.30, 60.22)	0.27
Diseases of musculoskeletal system in blood relatives	1.10 (0.38, 3.20)	0.86	1.00 (0.30, 3.33)	0.99	0.82 (0.24, 2.81)	0.76
Past disease or trauma of musculoskeletal system	1.23 (0.20, 7.66)	0.82	0.85 (0.20, 3.60)	0.82	0.85 (0.12, 6.11)	0.87
Top-level sport	2.44 (0.64, 9.33)	0.19	11.93 (1.89, 75.26)	0.0026	21.47 (1.84, 250.92)	0.002
Regular sporting activities	2.75 (0.70, 10.85)	0.14	0.85 (0.15, 4.84)	0.85	0.14 (0.02, 0.89)	0.018
Awareness of MSDs among dentists	0.99 (0.32, 3.06)	0.99	1.72 (0.53, 5.66)	0.37	1.12 (0.30, 4.13)	0.87
Considering the range of ergonomic education as sufficient			1.50 (0.39, 5.84)	0.56	0.81 (0.14, 4.67)	0.81
Whole model		0.74		0.47		0.04996

OR, odds ratio; CI, confidence interval.

**Table 5:** The multivariate analysis of the influence of the followed factors on the occurrence of lower back pain.

	1st Year		3rd Year		5th Year	
	OR (95% CI)	*p*	OR (95% CI)	*p*	OR (95% CI)	*p*
Gender (men)	3.42 (0.40, 29.49)	0.24	0.66 (0.10, 4.50)	0.67	0.25 (0.04, 1.67)	0.14
Age	0.87 (0.51, 1.46)	0.59	1.45 (0.79, 2.67)	0.19	2.16 (1.00, 4.65)	0.015
Height	0.84 (0.73, 0.96)	0.0068	0.95 (0.84, 1.07)	0.38	1.03 (0.91, 1.17)	0.64
Actual weight	1.13 (1.02, 1.25)	0.0098	1.00 (0.91, 1.10)	0.98	0.94 (0.85, 1.03)	0.17
Weight increase			0.52 (0.13, 2.07)	0.2	0.66 (0.18, 2.47)	0.43
Weight decrease			2.84 (0.58, 13.94)		0.27 (0.03, 2.29)	
General disease	1.34 (0.23, 7.93)	0.75	0.36 (0.05, 2.86)	0.33	1.13 (0.14, 9.22)	0.91
Chronic medication	1.39 (0.30, 6.35)	0.67	0.31 (0.05, 1.81)	0.18	1.14 (0.11, 12.04)	0.92
Dominant hand (right)	0.78 (0.10, 6.17)	0.81	0.18 (0.02, 1.95)	0.14	4.70 (0.36, 61.79)	0.22
Diseases of musculoskeletal system in blood relatives	0.53 (0.16, 1.69)	0.28	0.29 (0.08, 0.96)	0.037	1.97 (0.59, 6.60)	0.27
Past disease or trauma of musculoskeletal system	1.07 (0.13, 8.62)	0.95	1.12 (0.27, 4.61)	0.88	1.46 (0.29, 7.33)	0.65
Top-level sport	9.36 (1.88, 46.63)	0.003	0.56 (0.11, 2.94)	0.48	2.67 (0.51, 14.04)	0.24
Regular sporting activities	1.06 (0.25, 4.47)	0.93	2.22 (0.39, 12.75)	0.37	0.76 (0.16, 3.52)	0.72
Awareness of MSDs among dentists	0.72 (0.21, 2.51)	0.6	1.29 (0.40, 4.16)	0.68	3.91 (1.10, 13.88)	0.028
Considering the range of ergonomic education as sufficient			0.62 (0.16, 2.42)	0.49	0.85 (0.20, 3.56)	0.83
Whole model		0.071		0.29		0.15

OR, odds ratio; CI, confidence interval.

## Data Availability

The dataset is available upon request from the last author.
